# Temporally Coupled Coordination of Eye and Body Movements in Baseball Batting for a Wide Range of Ball Speeds

**DOI:** 10.3389/fspor.2020.00064

**Published:** 2020-06-26

**Authors:** Yuki Kishita, Hiroshi Ueda, Makio Kashino

**Affiliations:** ^1^Department of Information and Communications Engineering, School of Engineering, Tokyo Institute of Technology, Tokyo, Japan; ^2^NTT Communication Science Laboratories, Nippon Telegraph and Telephone Co., Kanagawa, Japan

**Keywords:** baseball batting, eye movements, head movements, hand-eye coordination, predictive saccades

## Abstract

We investigated the visuomotor strategies of baseball batting, in particular, the relationship between eye and body (head and hip) movements during batting for a wide range of ball speeds. Nine college baseball players participated in the experiment and hit balls projected by a pitching machine operating at four different ball speeds (80, 100, 120, 140 km/h). Eye movements were measured with a wearable eye tracker, and body movements were measured with an optical motion capture system. In the early period of the ball's flight, batters foveated the ball with overshooting head movements in the direction of the ball's flight while compensating for the overshooting head movements with eye movements for the two slower ball speeds (80 and 100 km/h) and only head rotations for the two faster ball speeds (120 and 140 km/h). After that, batters made a predictive saccade and a quick head rotation to the future ball position before the angular velocity of the ball drastically increased. We also found that regardless of the ball speed, the onsets of the predictive saccade and the quick head movement were temporally aligned with the bat-ball contact and rotation of the hip (swing motion), but were not correlated with the elapsed time from the ball's release or the ball's location. These results indicate that the gaze movements in baseball batting are not solely driven by external visual information (ball position or velocity) but are determined in relation to other body movements.

## Introduction

In hitting sports such as baseball, cricket, and tennis, players must make very accurate predictions about where and when the ball will come. Table tennis players, for example, demonstrate timing accuracies as precise as 2–5 ms (Bootsma and van Wieringen, 1990). Baseball batters also require accurate spatial bat control, to centimeter order precision considering the diameter of the bat. To make an accurate estimation of the ball trajectory, the gaze movement strategy is considered to be a critical factor because it affects the quality of the visual information gathered by the eye. Moreover, the gaze movement strategy tells us which visual information during a ball flight is important (Land and McLeod, 2000). In fact, many studies have indicated that visual strategies differ depending on the player's skill level (Land and McLeod, 2000; Rodrigues et al., 2002; Mann et al., 2013; Kishita et al., 2020).

Most of the studies on visual strategies of sports players have focused on what kind of visual information the players obtain and how they obtain it. The previous studies have revealed locations and events that are important for a good performance and how good players obtain such visual information. One visual strategy is predictive eye movements, wherein the eyes move to the locations where critical events will happen. In some hitting sports, players often make saccades to the locations where the ball will bounce in the future; e.g., this has shown to be the case in cricket (Land and McLeod, 2000; Croft et al., 2010; Mann et al., 2013), tennis (Williams et al., 1998), squash (Hayhoe et al., 2012), and table tennis (Land and Furneaux, 1997). The visual information from the place that the ball will bounce is considered to be useful for estimating the ball trajectory after the bounce. As well, other distinctive strategies, such as head tracking in cricket (Mann et al., 2013) and quiet eye in table tennis (Rodrigues et al., 2002), are considered to be effective ways of obtaining visual information.

However, gaze movements are sometimes affected by body movements and cannot be just for obtaining visual information. Laboratory studies suggest that body movements suppress gaze behavior. Neggers and Bekkering (2001) showed that gaze is anchored during reaching tasks, and such anchoring is not due to visual information being gathered. Furthermore, close temporal correlations between eye and hand movements have been demonstrated in several different visuomotor tasks (Sailer et al., 2000). In addition, the visual strategy may vary between hitting/intercepting the ball and just looking at the ball. It was shown that baseball players track the ball with smooth-pursuit eye movements when they watch it in flight but do not try to hit it (Bahill and LaRitz, 1984). In contrast, they use a saccade toward the impact position just before the bat-ball contact when they try to hit it (Kishita et al., 2020). This discrepancy in gaze movements indicates that the swing motion affects the visual tracking strategy.

Predictive eye movements toward the impact position such as what occurs in baseball batting (Kishita et al., 2020) are also observed in other hitting sports, including tennis (Williams et al., 1998), table tennis (Rodrigues et al., 2002), and cricket (Mann et al., 2013). What is interesting here is that the time from the landing of saccades to the bat-ball contact is too short for the swing motion to reflect the visual information due to the delay of visuomotor processes (Kishita et al., 2020). This fact leads us to consider other functional significances of this gaze shifting behavior besides obtaining visual information. Eye movements are known to convey position information on objects in the eye-centered coordinates used for motor planning (Buneo and Andersen, 2006). The same mechanism may be at work when catching and hitting in interactive ball sports. Thus, for a comprehensive understanding of gaze behavior during sports, not only is the strategy of acquiring visual information important, so is its relationship with body movements. In the case of baseball batting, the torso starts rotating first, and then a bat is accelerated. Since the order of these sequential movements is maintained in most cases, the timing of the torso rotation seems to reflect the expected time of ball arrival.

In this study, we investigated how differences in ball speed affect the visual strategies of baseball batters. Specifically, we measured eye, head, and hip movements of nine college baseball players during a batting task conducted under four different ball-speed conditions (80, 100, 120, and 140 km/h). The data collected on the eye and head movements were used to investigate the visual strategies. By measuring both eye and head movements, the contributions of the eye and head movements to the overall gaze movements can be calculated. The data collected on the hip rotation was used to clarify the relationship between the visual strategy and swing motion. These data reveal how the batters moved their gaze for different ball speeds and how eye and head movements each contributed to gaze shift changes. Moreover, the results provide a better understanding of the mechanism of gaze control in hitting tasks, such as whether the visual strategy is determined only by external factors (visual information: e.g., ball position and velocity) or by internal factors (body movements).

## Methods

### Participants

Nine college baseball field players (five right-handed batters and four left-handed batters, aged 20–22 years: *M* = 20.78, *SD* = 0.83) participated in this experiment. The participants play in the Tokyo Big6 Baseball League, which is one of the best college baseball leagues in Japan. All the participants provided written informed consent before the experiments. This study was approved by the Ethics and Safety Committees of NTT Communication Science Laboratories and was under the Declaration of Helsinki.

### Apparatus

Eye and head movements were measured with the same apparatus used in Kishita et al. (2020). Eye movements were recorded monocularly with a wearable eye tracker (Pupil headset, Pupil Labs GmbH, Germany). Left-handed batters' right eyes and right-handed batters' left eyes were recorded to reduce asymmetry. The sampling frequency of the eye camera and scene camera (which recorded the view from in front of the batter's head) were 200 and 120 Hz, respectively. Consequently, the eye position data were recorded at a sampling frequency of 200 Hz. The ball positions were manually digitized from thinned-out scene camera images and resampled to match the sampling frequency of the eye position data. Head and hip movements were measured at a sampling frequency of 240 Hz with an optical motion capture system (Optitrack, NaturalPoint, Inc., the U.S.). Participants wore a helmet that was tightly fixed to their heads with a headband and chin strap. Reflective markers were attached to the helmet for the optical motion capture system to measure the head movements. A waistband with reflective markers attached to it was worn to measure the hip movements. The participants hit balls projected by a three-wheel pitching machine (Pitch 18, Nishino-Machinery Corporation, Japan). The distance between the ball acceleration point of the pitching machine and the bottom of home plate was 16.7 m. The pitching machine was adjusted so that the ball would pass the center of home plate at the level of the participants' waist (i.e., the center of the strike zone). The time of bat-ball contact was detected from videos recorded with a high-speed camera at a sampling frequency of 300 Hz. The ball-release timing was obtained from a photosensor attached to the pitching machine. The devices were synchronized using LED lights that flashed at the start of motion-capture recording. The LED lights were captured by the high-speed camera recording the bat-ball contact and the scene camera of the eye tracker.

### Procedure and Design

The experiment began with the calibration of the eye tracker using the manual marker calibration provided by the operating software. The eye camera and the scene camera images were monitored in real time, and recalibration was performed whenever it was needed.

Participants were instructed to hit the ball strongly toward the center of the field. The pitch type was fastball only. The ball speed was set to 80, 100, 120, or 140 km/h. The mean ball speeds for each condition, obtained from two photosensors on the pitching machine, were 78.8 (*SD* = 0.85), 102.5 (*SD* = 1.64), 123.3 (*SD* = 2.02), and 142.5 (*SD* = 2.33) km/h. Each ball-speed condition was tested in a separate block of trials. In each block, five participants started with the slowest ball condition and proceeded to the faster conditions, while the other four participants started with the fastest condition and proceeded to the slower conditions. Before starting each block, participants practiced several times to get used to the ball speed of the new block. In each block, the trials were repeated until the number of the bat-ball contacts reached 11–13 times.

### Data Analysis

**Figure 1:**
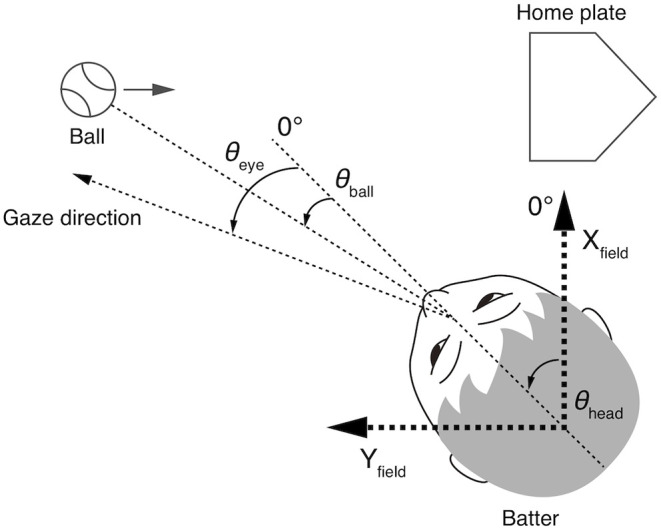
Geometry of parameters used in the analyses from Kishita et al. (2020). The Y_field_ axis is parallel to the pitcher-home plate direction, and the X_field_ axis is orthogonal to the Y_field_ axis. The origin of the X_field_-Y_field_ coordinates is at the center of the head. The head directions (θ_head_) is expressed in the X_field_-Y_field_ coordinates, and the eye direction (θ_eye_) and the ball direction (θ_ball_) are relative to the head direction.

The eye and head movement parameters were processed and expressed using the same methods as in the previous study (Kishita et al., 2020). The parameters and the coordinate system are illustrated in Figure 1. The Y_field_ axis was parallel to the pitcher-catcher direction and the X_field_ axis was perpendicular to the Y_field_ axis. The origin of the coordinate system was the center of the head. Thus, the position of the origin moved with the translational motion of the head. The data of the left-handed batters were inverted to fit this coordinate system.

The head direction (θ_head_) was defined as the angle between the X_field_ axis and the median plane of the head. The head direction was calculated from the reflective markers on the helmet. The eye position (θ_eye_) was represented as the angle between the head direction and the gaze direction, and the ball direction (θ_ball_) was represented as the angle between the head direction and the ball direction in the horizontal plane. Here, we call the direction of gaze relative to the X_field_-Y_field_ coordinates the gaze direction (θ_head_ + θ_eye_) to distinguish it from the eye position (θ_eye_), which is the direction of the gaze relative to the head direction. The eye and ball position data were corrected for the effects of lens distortion and head tilt. The eye position data were smoothed with a second-order Savitzky-Golay filter with a window size of nine points. The hip direction (θ_hip_) was defined as the angle between the median plane at the height of the waistband and the X_field_-axis. The hip movements obtained from the motion capture data were smoothed by with a second-order Savitzky-Golay filter with a window size of fifty points. All movement data were resampled to match the sampling frequency of the eye position data (200 Hz).

Data from the trials in which the head direction or the eye position at the time of ball release deviated by more than 3 *SD* compared with the other trials conducted under the same conditions were excluded from the data sets. The number of data for each speed condition used for analysis was from 8 to 13 trials. To elucidate the general characteristics of baseball batters, we analyzed the mean data of all participants obtained from the mean data of each player.

### Statistical Analyses

**Figure 2:**
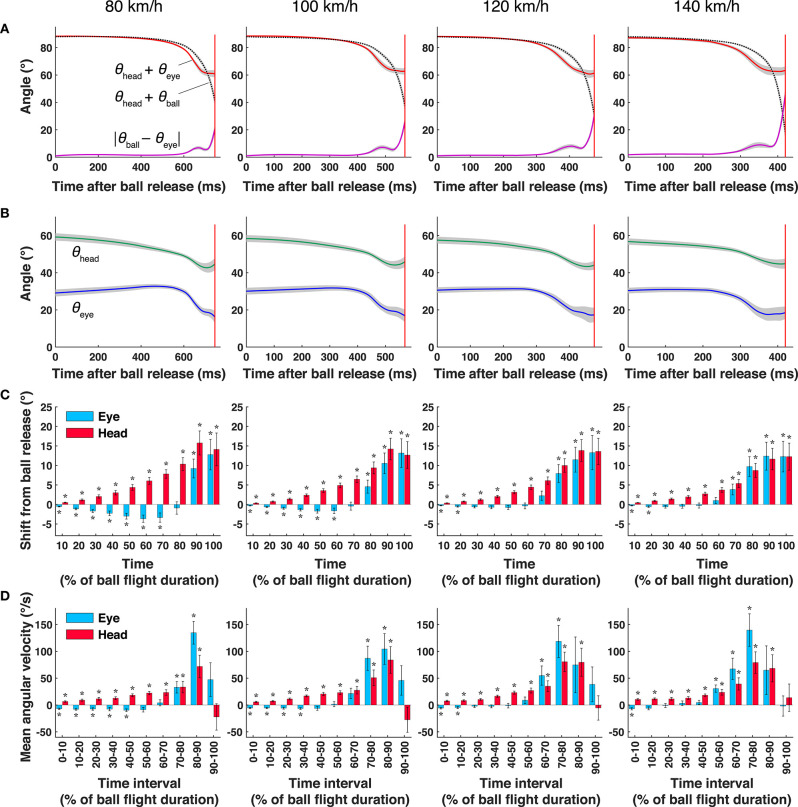
Mean gaze direction, eye position, and head direction at different ball speeds. **(A)** Gaze direction (θ_head_ + θ_eye_: red line), ball direction in field coordinates (θ_head_ + θ_ball_: black dashed lines), and difference between gaze direction and ball direction (|θ_eye_ – θ_ball_|; purple lines). **(B)** Mean head direction (θ_head_: green lines) and eye direction (θ_eye_: blue lines). The shaded areas represent the standard error. **(C)** The amount of eye and head movements from ball release to each 10% of the ball's flight duration. **(D)** Mean angular velocity of eye position and head direction within each time interval. Each interval (%) was obtained by dividing the total time from ball release to bat-ball contact into 10 equally spaced bins. The error bars represent the standard error, and the asterisks represent the results of *t*-tests (*p* < 0.05); the mean values are significantly larger or smaller than zero. The red vertical lines show the mean bat-ball contact time.

For each condition, the contributions of eye and head movements to the ball tracking were assessed by two-tailed one-sample *t*-tests applied to the amount of eye and head movements from the ball release to each 10% of the ball's flight duration (Figure 2C) and to the mean angular velocities of the eye and head movements during each 10 equally divided time interval (Figure 2D). The sample size for each test was nine, corresponding to the number of participants.

For each participant and condition, we expressed the time and the ball position when the eye movements reached the peak velocity (representing the saccadic eye movement) by (1) the time from ball release, (2) the time before the bat-ball contact, and (3) the ball's distance from home plate (Table 1). These values were obtained from the mean data of each participant. To examine which events are in a temporally coupled relationship with the eye movements, we performed a one-way repeated-measurements ANOVA on each data set aligned at the different events. Ryan's method was used as a *post-hoc* test with an alpha level of 0.05.

**Table 1:** The time when the velocity of eye movements peaked and the ball position at that time.

	**Release aligned time (ms)**		**Impact aligned time (ms)**		**Ball position (m)**	
**Ball speed**	**Mean**	***SD***	**Mean**	***SD***	**Mean**	***SD***
80	642	23.3	−108	26.6	2.38	0.57
100	465	37.5	−107	37.5	3.09	1.08
120	373	38.0	−105	38.8	3.62	1.33
140	316	32.8	−104	35.3	4.08	1.35

*The mean values and the standard deviations were calculated from the mean values of each participant. The release aligned time represents the time after ball release, and the impact aligned time represents the time before the bat-ball contact. The ball position shows the distance between the ball and home plate at the time when the eye movement velocity is at its peak*.

## Results

### Strategies for Tracking at Different Ball Speeds: Different Amounts of Eye and Head Movement

Figure 2A shows the mean gaze direction (θ_head_ + θ_eye_: red lines), mean ball direction (θ_head_ + θ_ball_: black dotted lines), and their difference (i.e., the mean error, |θ_ball_ – θ_eye_|: purple lines) in the X_field_-Y_field_ coordinates. As can be seen from the magnitude of the error, for all ball-speed conditions, batters were able to track the ball accurately for a while after the ball was released. However, the amounts of eye and head movement (blue and green lines in Figure 2B, respectively) varied during the tracking period and depending on the ball-speed conditions. To illustrate these differences, we plot the amount of eye and head movements from ball release to each 10% of the ball's flight duration (Figure 2C) and their mean angular velocities during each time interval (Figure 2D). The percentage on the x-axis shows time (% of the ball's flight duration), and a positive value on the y-axis indicates the direction of the ball's flight.

First, although the final displacement of the eye position and head direction were almost the same for all ball-speed conditions (range of 12–14° at 100% in Figure 2C), the amounts of eye and head movement differed greatly between the early and late stages of tracking. In the early period (Figures 2C,D), when the ball moved through a smaller visual angle, batters mainly rotated their head in the direction of the ball's flight. On the other hand, eye movements increased in the later period (Figures 2C,D), when the angular velocity of the ball dramatically increased (Figure 2A). As can be seen from the velocity profile in Figure 2D, the eye movements exceeded 100°/s at some time in the latter half of the flight, which indicates that saccadic eye movements took place. In addition, Figure 2A shows that these saccades were predictive because they landed on the future location of the ball, and that the error between the eye positions and ball directions (|θ_ball_-θ_eye_|) became large during this saccade period.

The amounts of eye and head movement depended not only on the phases of tracking but also on the speed conditions. During the first half of the two lower speed conditions (80 and 100 km/h in Figure 2D), we observed that the head and eyes rotated in opposite directions; the head overshot the ball in the direction of the ball's flight, while the eyes moved in the opposite direction to compensate for the overshoot component. In contrast, under the faster ball-speed conditions (120 and 140 km/h in Figure 2D), the compensatory eye movements appeared only in the early (0–20%) period. For all speed conditions, the amount of eye movement in the direction of the ball's flight became significant when the angular velocity of the ball abruptly increased (about 60–90% in Figure 2D).

### Temporal Relationship Between Eye and Body Movements

**Figure 3:**
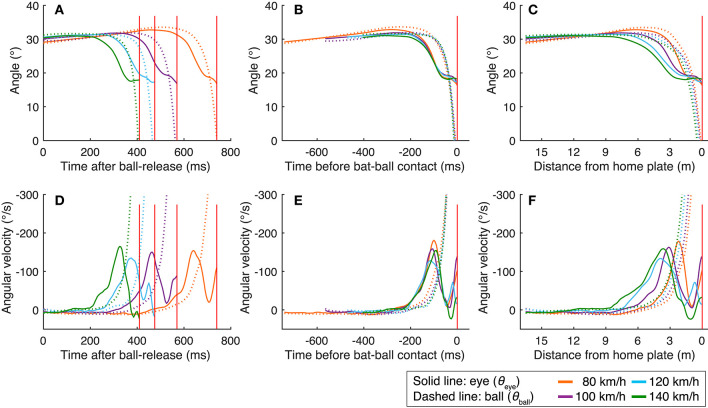
Mean eye positions and eye velocities aligned to three different events. The eye positions are plotted as a function of time after ball release **(A)**, time before bat-ball contact **(B)**, and distance of the ball from home plate **(C)**. The angular velocities are shown in the same way: as a function of time after ball release **(D)**, time before bat-ball contact **(E)**, and distance of the ball from home plate **(F)**. The red vertical lines show the mean time or location of the bat-ball contacts. The colors of the line indicate ball speed. The dotted lines show the ball's angular position or velocity.

To examine what triggered the eye movements, especially the predictive saccades, we plotted the mean angle of the eye position and the mean angular velocity of the eye movements against the time after ball release (Figures 3A,D), time before bat-ball contact (Figures 3B,E), and against the ball's distance from home plate (Figures 3C,F). As is obvious from the figures, the saccade onsets were aligned with the time of the bat-ball contact at all ball speeds. To statistically assess this, we obtained the times and the ball positions when the eye movements reached the peak velocity aligned with different events (ball release, bat-ball contact, distance of the ball from home plate: Table 1), and applied a one-way repeated-measures ANOVA to each data set. A significant main effect was found for the time of the peak eye velocity aligned with ball release [ANOVA: *F*_(3,24)_ = 827, *p* < 0.01; *post hoc t*-tests: *p* < 0.01 for all pairs] and for the ball position at the time of peak eye velocity [ANOVA: *F*_(3,24)_ = 15.65, *p* < 0.01; *post hoc t*-tests: *p* < 0.01 for all pairs except for 100 vs. 120 km/h (*p* < 0.1) and 120 vs. 140 (*p* < 0.1)] but not for the time of the peak eye velocity aligned with bat-ball contact [*F*_(3,24)_ = 0.171, *p* = 0.915]. This indicates that the predictive saccades were not triggered by some event in time or space after the ball was released, but by prediction of the time of bat-ball contact, which indicates a temporal relationship between eye and other body movements.

**Figure 4:**
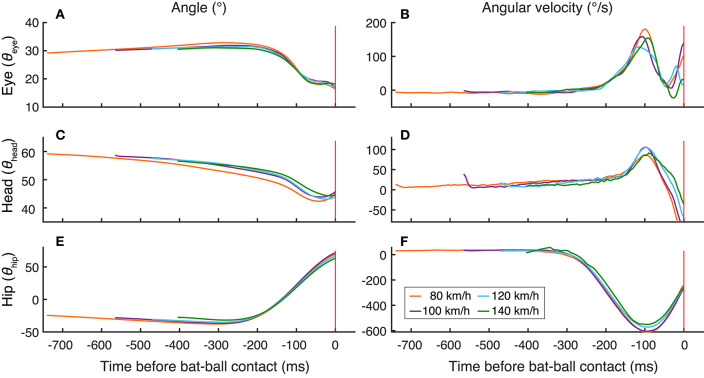
Temporal changes of the mean positions of the eye **(A)**, head **(C)**, and hip **(E)** and the mean velocities of the eye **(B)**, head **(D)**, and hip **(F)**. All the data are aligned at the time of bat-ball contact. The colors of the lines indicate the ball-speed condition. The red vertical lines show the bat-ball contact time.

Similar results can be seen in the plots of head and hip positions and movements aligning at the time of bat-ball contact (Figure 4). The bat-ball contact and hip movement were temporally aligned for all ball-speed conditions. In addition, the hip movement and the eyes and head movements were also neatly aligned. These results provide evidence of a very close temporal connection between eye and body movements in baseball batting.

## Discussion

In the present study, we investigated baseball batters' visuomotor strategies for different ball speeds. The common characteristics for all ball-speed conditions were that batters foveate the ball for a while after it is released, mainly by using head rotations, and then use a saccade and a quick head rotation toward the future position of the ball. These characteristics are consistent with our previous study on professional baseball players (Kishita et al., 2020). In addition, by using conditions with different ball speeds, we revealed two interesting characteristics that (1) overshooting head movements in the direction of the ball's flight and compensatory eye movements in the opposite direction were observed for slower than familiar ball speeds and (2) eye movements were controlled in a temporal connection with bat-ball contact.

### Eye and Head Movements in Relation to the Speed of the Ball

The batters who participated in the current study usually bat against pitches corresponding to the two faster ball conditions (120 and 140 km/h). Therefore, it is plausible that they usually adopt the strategy of tracking the ball using head rotations without eye movements for the initial ball trajectory (or for small visual changes), as was observed in the faster ball conditions. Head tracking has been used in many ball sports and similar situations, and its benefits have been discussed in relation to, e.g., catching the ball (Oudejans et al., 1999; Zaal and Michaels, 2003), basketball jump shooting (Ripoll et al., 1986), cricket batting (Mann et al., 2013), and baseball batting (Fogt and Zimmerman, 2014; Higuchi et al., 2018; Kishita et al., 2020). Studies on the visual-motor system have also suggested the benefits of head tracking. It has been pointed out that motor planning in visual-motor tasks is performed in the egocentric coordinate system, which is crucial for the visuomotor system (see e.g., De Wit et al., 2012). Mann et al. (2013) mentioned that keeping the direction from the head to the target constant may be advantageous for representing the target position in an egocentric coordinate system. Therefore, baseball batters may try to track the ball with head movements as much as possible while maintaining the ball's direction relative to the head in order to represent the ball in a head-centered coordinate system and to facilitate accurate motor planning.

In the two slow-speed conditions (80 and 100 km/h), overshooting head movements in the direction of the ball's flight and compensatory eye movements in the opposite direction were observed during the first half period (0–50%, in Figure 2D). However, it seems that different functions work in the early (0–20%) and late (20–50%) stages. Shortly after the ball is released (0–20%), the function appears to be a typical vestibular eye reflex (VOR), where the eyes and head move in opposite directions at approximately the same speed. This was seen under all speed conditions. In contrast, in the next period (20–50%), the speed of the head movement gradually increases compared to the speed of the eye movement while tracking the ball. In this period, the position of the ball acquired on the retina is thought to be reflected in the eye movements. This gaze strategy that tracks moving objects using a combination of eye and head movements is called combined eye-head tracking (CEHT). CEHT is considered to be realized by adjusting both smooth pursuit and VOR signals (Lanman et al., 1978; Huebner et al., 1992). In this sense, the head tracking seen under the faster ball-speed conditions (120 and 140 km/h) can also be regarded as a type of CEHT (Huebner et al., 1993).

Why did the batters produce almost the same head movements regardless of the speed of the ball and adjust their gaze direction by using eye movements? This is probably because their head movements for tracking the ball thrown by the pitcher were acquired during the course of a long period of training and are difficult to change. In other words, eye movements are more agile and more accurate for making small gaze adjustments. Our findings suggest that for sudden (unexpected or unfamiliar) changes in target positions, it is easier and more accurate to adjust the gaze position by using eye movements than by using head movements.

### What Determines the Visual Tracking Strategy in Baseball Batting?

Our results indicate that batters' visual tracking strategy is constrained not only by the ball's trajectory, but also by other body movements that affect the batting action. First, the visual tracking behaviors (i.e., eye and head movements) have strong temporal coupling with the swing motion, regardless of the ball's speed. The onset of the predictive saccade and quick head rotation was precisely aligned with the time of bat-ball contact and hip rotation. The same temporal alignment would not occur if the specific ball speed or position were used as a reference for the eye and head movements. Second, the fact that the saccade was predictive also indicates that eye movements were not solely determined by the visual information about the ball. When tracking a moving object, saccades are often triggered by retinal slips due to the speed limitation of smooth-pursuit eye movements (de Brouwer et al., 2002). However, here, the saccades started when the ball was much slower than the maximum speed of smooth-pursuit eye movements (Figures 3D–F). This also implies that the batters' made the saccades actively in response to the ball's movement and predicted when the bat-ball contact would take place.

One possible reason why visual tracking behavior is determined by the temporal relationship with movements of other body parts is that the maximum time available for gathering visual information for hitting depends on the timing of the bat swing. Since the goal of batting is to bring the bat into contact with the ball, the batter needs to adjust his/her swing to the movement of the ball. However, once the swing starts, the delay of the visuomotor processes restricts the amount of visual information that can be reflected in the swing motion, so it is not surprising that the visual strategy depends on the swing motion. However, this does not answer the question of why batters shift their gaze position to the future ball position by using the predictive saccades and quick head rotation.

### Why Shift the Gaze to the Impact Position in Time With the Swing Motion?

One simple interpretation for shifting the gaze position in advance is to obtain visual information at the impact position. Rodrigues et al. (2002) mentioned that table tennis players stabilize their head and eyes just before the racket makes contact with the ball in a forehand stroke. However, from the viewpoint of how long it takes the brain to process visual information, it has also been pointed out that even if visual information at the time of bat-ball contact can be obtained, it is difficult for the swing motion to reflect that information quickly enough in movements online (Bahill and LaRitz, 1984; Kishita et al., 2020). In the present study, the gaze and ball positions overlapped about 50 ms before the bat-ball contact (Figure 2A), which also seems too short for the ball position information to be reflected in the swing motion. Furthermore, visual occlusion experiments on baseball batting have confirmed that visual occlusion occurring 150 ms before arrival of the ball has no significant effect on batting performance (Higuchi et al., 2016). For these reasons, if the gaze moves to the impact position in order to obtain visual information, the information gained there is likely used for learning in the future, not for online visuomotor responses.

Another possible reason for gaze shifting before the ball comes is that batters utilize gaze to build a representation of predicted impact positions for motor planning. The look-ahead fixation not only gets visual information but also passes the location of the target of motor planning (Land and Tatler, 2009). In this process, an efference copy of eye movements provides the eye position information for motor planning, which is critical for calculating the distance between the target and limb (Lewis et al., 1998). Importantly, the efference copy signal can be used immediately, i.e., without delay, which ensures that it can be used in the current motion. Exact temporal coupling of eye and body movements has also been observed in simple reaching tasks, which are thought to result from sharing of the nervous system between eye and body movements (see, e.g., Sailer et al., 2000). We suggest that the same may occur in baseball batting.

## Conclusion

We investigated the eye and body movements of baseball players batting against a wide range of ball speeds. We found that their visual strategies exhibited two characteristics: first, in the early period of the ball's flight (when the change in the visual angle is small), batters adopt different visual strategies, i.e., tracking the ball by overshooting head movements with compensatory eye movements in the opposite direction when it's slow (80 and 100 km/h) or by using head movements when it's fast (120 and 140 km/h). These different strategies are probably the result of a long period of training involving tracking balls with head movements and responding to other sudden speed changes with eye movements. These results provide insights into how players use their eye and head movements depending on the ball's trajectory and body movements in many ball sports. In addition, they suggest the possibility of developing new vision training that also uses head tracking, rather than conventional vision training that aims to accurately track the target only by eye movements, as represented by dynamic visual acuity training. The second finding is that the timing of the major gaze shifts (predictive saccade and rapid head movement) is determined by the timing of the bat-ball contact, regardless of the ball's speed. This means that the visual strategy is determined not only by the visual information associated with the movement of the target, but in some situations, it is also determined in relationship with body movements. Since the focus here was on clarifying general visuomotor strategies of baseball batters, the batter's level- or type-specific characteristics may not have been fully captured. However, our findings may reveal not only the characteristics of baseball batters but also the basic characteristics of human visuomotor coordination under such extreme conditions: interaction with an object moving at a very high speed by using unconstrained eye and head movements.

## Data Availability Statement

The datasets generated for this study are available on request to the corresponding author.

## Ethics Statement

The studies involving human participants were reviewed and approved by NTT communication science laboratories. The patients/participants provided their written informed consent to participate in this study.

## Author Contributions

YK, HU, and MK conceived, designed the experiments, and interpreted the data. YK and HU performed the experiment. YK conducted the data analysis and drafted the manuscript. HU and MK edited and revised the manuscript, and approved the final version.

## Conflict of Interest

MK and HU were employed by NTT Communication Science Laboratories, Nippon Telegraph and Telephone Corporation. The remaining author declares that the research was conducted in the absence of any commercial or financial relationships that could be construed as a potential conflict of interest.
